# A coastline generalization method that considers buffer consistency

**DOI:** 10.1371/journal.pone.0206565

**Published:** 2018-11-01

**Authors:** Hui Yang, Lin Li, Hai Hu, Yanlan Wu, Hui Xia, Yu Liu, Shudong Tan

**Affiliations:** 1 School of Resource and Environment Sciences, Wuhan University, Wuhan, China; 2 Collaborative Innovation Centre of Geospatial Technology, Wuhan University, Wuhan, China; 3 School of Resources and Environmental Engineering, Anhui University, Hefei, PR China; 4 National Marine Data and Information Service(NMDIS), Hedong District, Tianjin, PR China; University of Maine, UNITED STATES

## Abstract

Coastlines are the boundary between the ocean and land and are an important line feature in spatial data. Coastlines must be adapted via map generalization when they are stored in multi-scale spatial databases. This article presents a new method of coastline generalization that considers the buffer consistency from the original coastline to the generalized coastlines. This method uses the geographical distance field to identify the feature points and bends that influence the buffer consistency from the original coastline to the generalized coastline, and the process is also used to simplify the bends and maintain the concave and convex characteristics in the generalization. This method is compared to the bend-simplification method, and the results indicate that the proposed method can preserve the shape characteristics of coastlines. Moreover, the seaward buffer boundaries from the original coastline to the generalized coastline are consistent when the distance is greater than the tolerance of the coastline generalization.

## Introduction

**Fig 1:**
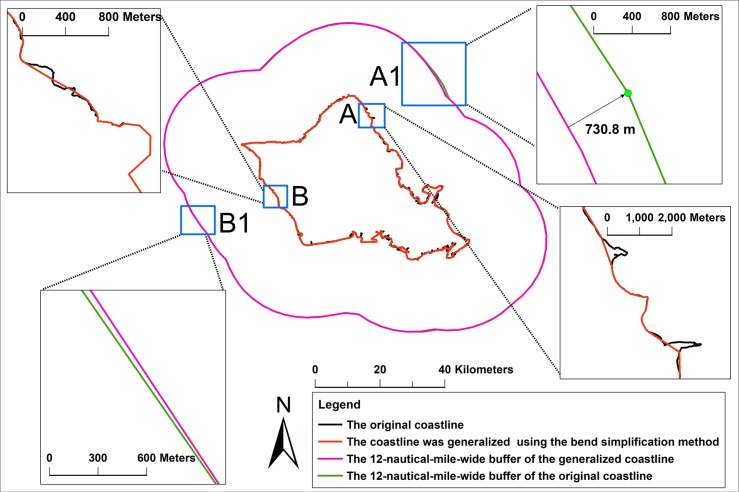
Twelve-nautical-mile-wide buffer created from the original coastline (1:75 K) and the 1:200 K coastline. The generalized coastline (1:200 K) was generated from the coastline (1:75 K) at a threshold of 1000 m using a bend simplification method. The rectangles show the effects of local amplification of the overlay comparison.

Coastlines are the boundary between the ocean and land, and they are often used in navigation, marine change monitoring, and maritime delimitation [1–3]. In particular, in maritime delimitation, when the coastline is used as a natural baseline, the coastline is the basis for the calculation of maritime boundaries, and the use of different coastline scales will directly affect the resulting maritime boundaries. Fig 1 shows the error in the calculation of maritime boundaries caused by the use of different coastline scales. For the calculation of twelve-nautical-mile territorial waters, when the coastline (1:75 K) and the generalized coastline (1:200 K) are used as the basis to calculate the territorial sea boundary, the boundaries of territorial waters were generally similar, and the partial boundary deviation is small (see rectangles B and B1 in Fig 1). However, coastline generalization (see rectangle A in Fig 1) will also cause large deviations in the boundaries of territorial waters, of which the maximum deviation can reach 730.8 meters (see rectangle A1 in Fig 1). This deviation may become larger when the principle of land priority is applied to generalize the coastlines for navigation safety. This is because coastline generalization using the principle of land priority will enlarge land area and shrink the ocean area. Therefore, when studying the coastline generalization method, it is necessary to consider differences in the spatial-analysis results of coastlines before and after generalization for some applications of coastlines (in particular, when the coastline needs to be used as a basis for spatial analysis, such as in maritime delimitation).

The method of line generalization is often applied to coastline generalization. Numerous techniques have been developed in the literature, such as the Douglas-Peucker method [4], topological objective methods [5, 6], the area-preserving method [7–9], the scale-specificity method [10–19], the shape-preserving method [20, 21] and so on.

The Douglas-Peucker algorithm is the most widely used method of line simplification [22–25]. However, this method has several problems, such as starting-point dependency, self-intersection, and shape distortion [13, 26–28]. The Douglas-Peucker method has been improved and expanded in many studies [22, 23, 29–36]. For example, Saafeld [33] proposed a method that uses a dynamically updated convex-hull data structure to detect and eliminate topology conflicts, thus overcoming the self-intersection issue in the Douglas-Peucker method. However, the Saalfeld method of line compression mainly involves data storage, which is different from generalization. The Douglas-Peucker algorithm does not produce optimal results for generalization of natural features such as coastlines [1, 11, 13, 26, 37].

Topological objective methods [5, 6] primarily focus on retaining the original relationships of connectivity and containment by using simplified features.

The main objectives of the area-preserving method [7–9] are to preserve the area of a polygon as much as possible during the polygon-boundary-simplification process and improve the quality of the polygon-boundary simplification in terms of its position and area error.

The scale-specificity method [10–19] structures the mathematical relationship between the generalization tolerance and the scale change to simplify a boundary. For example, the Radical Law that was proposed by Töpfer and Pillewiser [16] provides a formula to calculate how many instances of a feature should be retained from the original map to the target scale. Li and Openshaw [13] proposed a natural principle method that was based on the type of natural generalization process that can be observed in our daily life. When an object leaves an observer’s view, the image of the object becomes smaller. The details of this object become more difficult to discern, and the image of the object is considered as being generalized. This universal principle is called the “natural principle” by Li and Openshaw, which can be expressed as follows: for a given target scale, all the details of a geographical object (feature) beyond a certain tolerance cannot be presented and can be ignored. The natural principle method uses the ratio of the source scale to the target scale and the target map resolution to define a minimum visual object (SVO) window, arranges the SVO window along the line feature, and merges all the vertices that fall within the SVO window into a single vertex. Perkal's ε-circle rolling algorithm[17] calculates the generalization tolerance ε from the target scale and the line weight, generates a circle or wheel with a diameter of ε, and rolls it on each side of the polyline to identify the ε-convex portions. The enveloping zones that formed in this manner are also called boundary zones [11], which identify what portion of the line should be simplified.

The main objective of the shape-preserving method [20, 21] is to preserve the shape characteristics of the original line as much as possible. For example, Wang and Muller [21] proposed a coastline-simplification method that was based on the shape characteristics of bends, and Lewin et al. [20] proposed a simplified method through shape evolution.

Other methods, such as the map-generalization method [1, 38, 39], are based on feature semantics, and the generalization decision is made at a higher semantic level. Visvalingham and Whyatt [40] proposed a line simplification method that was based on the effective area to complement the Douglas-Peucker algorithm. Guilbert [41] proposed a method that was based on the B-spline snake model, which considers the limitations of the application to realize the visualization of the map, such as the accuracy, readability, and security.

Generally, these methods produce the best possible results based on their own objectives. However, these methods do not consider the differences in the spatial-analysis results of coastlines before and after generalization, such as buffer-consistency issues. Therefore, this paper proposes a coastline-generalization method that considers the buffer consistency. This method can both preserve the shape characteristics of a coastline and provide a radius for the buffer analysis under which the buffer-analysis result between the original coastline and the generalized coastline is consistent.

This article is organized as follows. First, we analyze the main factors that affect the buffer consistency. A coastline-generalization method that is based on the source point tracking of buffer boundaries is then presented. Finally, coastlines that are generalized at different scales by using a different method are analyzed to validate the results.

## Methodology

### Main factors that affect the buffer consistency

**Fig 2:**
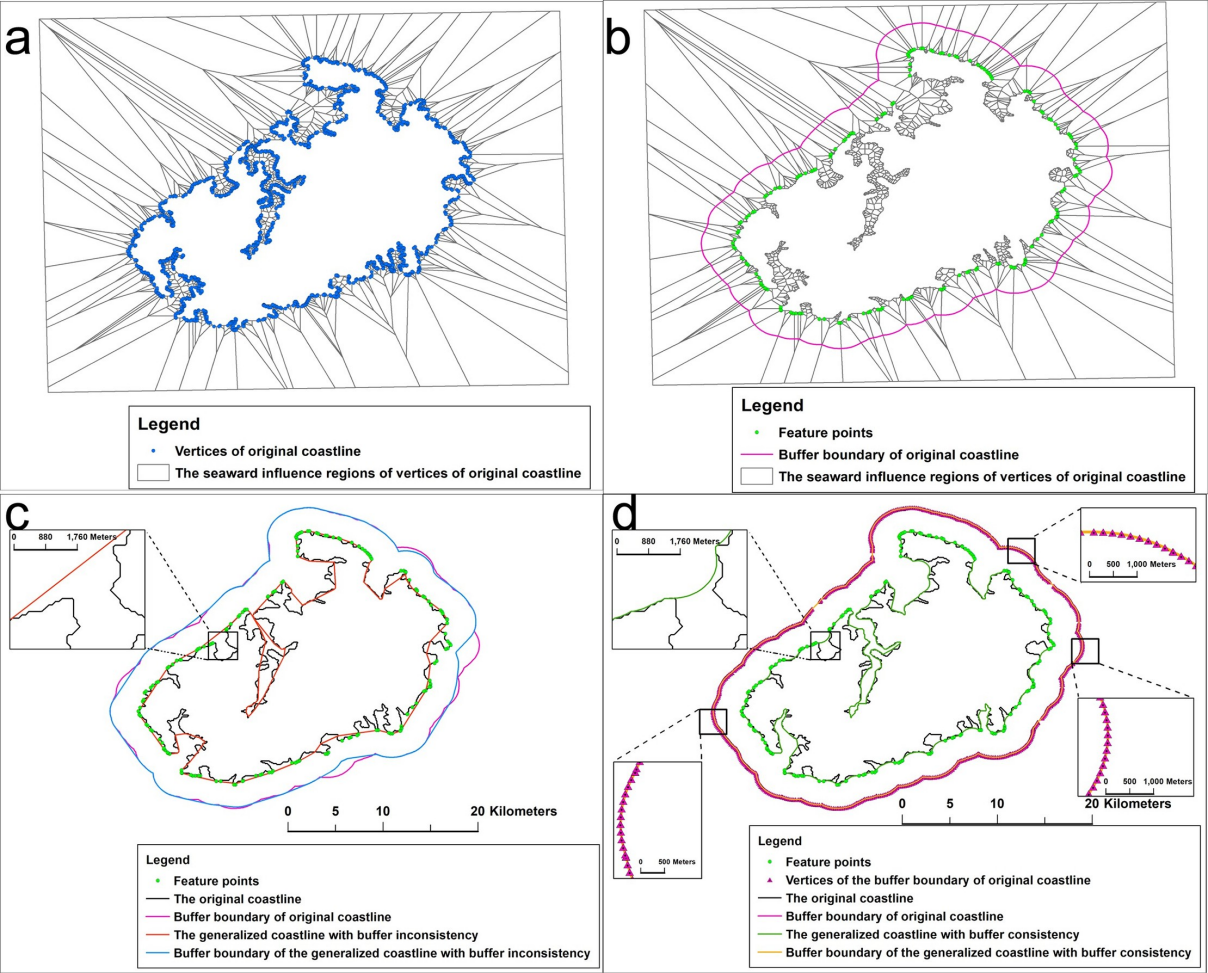
Comparison between the generalized coastlines with buffer consistency and inconsistency. (a) Seaward influence regions of the vertices of the original coastline; (b) vertices that the seaward influence regions reached or that were beyond the buffer boundary (a 3000-m-wide buffer); (c) 3000-m-wide buffer that was created from the generalized coastline (1:500 K) with buffer inconsistency; (d) 3000-m-wide buffer that was created from the generalized coastline (1:500 K) with buffer consistency.

The generation of a coastline's buffer is mainly affected by the shape of the coastline and the region of influence of the vertices. Fig 2 shows the differences between generalized coastlines with buffer consistency and inconsistency. The coastlines have the following main differences:

1. The generalized coastline with buffer inconsistency lost numerous feature points that the regions of influence reached or that were beyond the buffer boundary (Fig 2C).
2. The concavity and convexity of the generalized coastline with buffer inconsistency changed from that of the original coastline, and the deleted portions changed from bends to straight lines (Fig 2D).

The following key issues must be addressed to consider the buffer consistency from the original coastline to the generalized coastline:

1. The feature points that the regions of influence reach or that are beyond the buffer boundary must be identified.
2. The shape characteristics should be retained, and the displacement that is caused by bend simplification should be reduced, that is, the concavity and convexity of the coastline must be maintained when bends are deleted.

Therefore, a coastline-generalization method that is based on the source point tracking of buffer boundaries (SPTBB) is proposed to address these issues.

### Theory of coastline generalization based on the source point tracking of buffer boundaries

**Fig 3:**
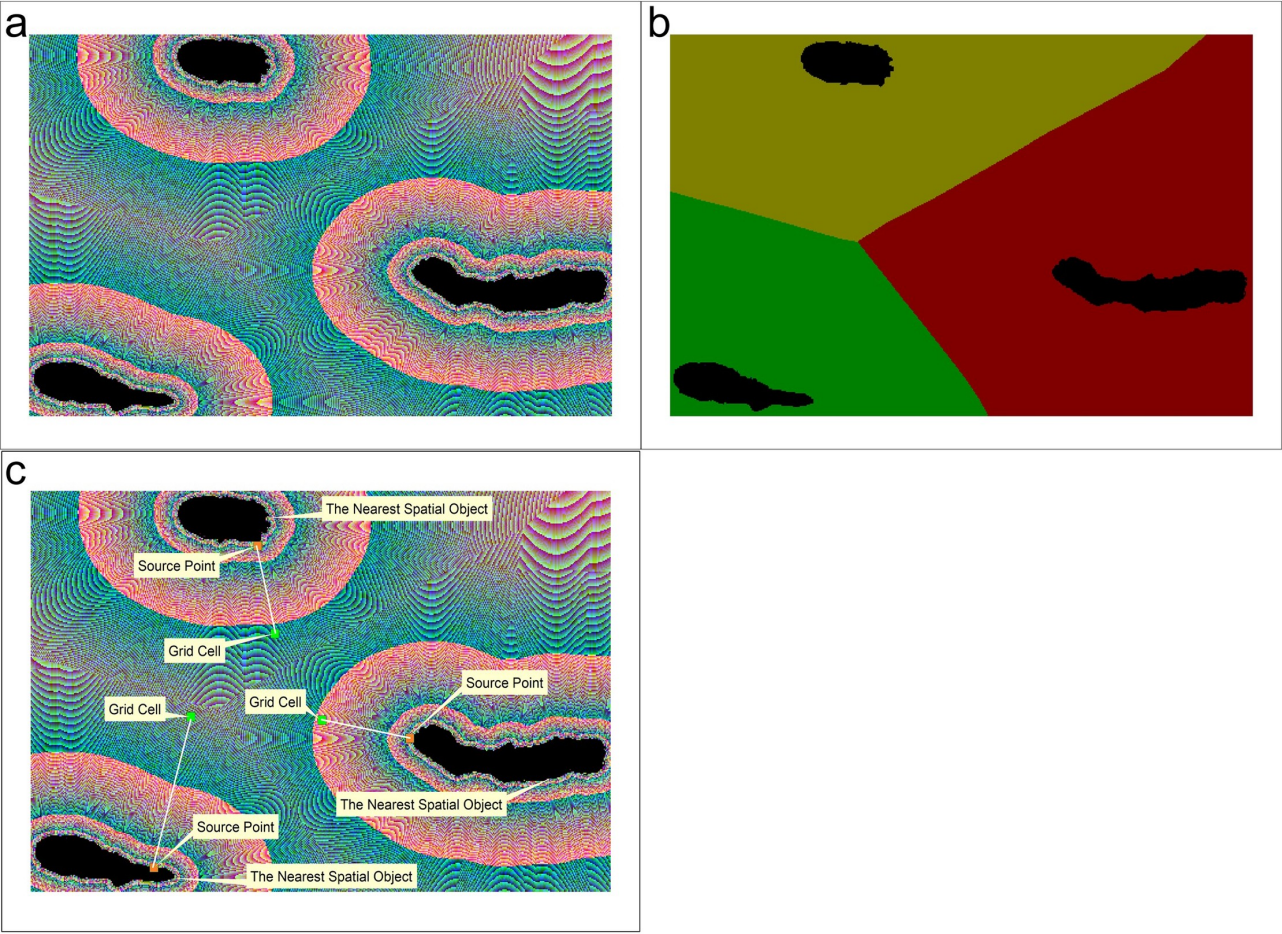
Composition of the geographical distance field. Matrices of the (a) distances, (b) source objects, and (c) source coordinates.

The geographic distance field is the product of geographic distance transformation. Geographic distance transformation uses multiple spatial matrices to model the influence of spatial entities on the surrounding space[42]. In the process of geographical distance transformation, three raster matrices are created. These matrices are used to store the distance, source-object and source-point information of each grid. The distance is the minimum geodesic distance from the center of the grid to the nearest spatial object (Fig 3A). The source object is the nearest object that is used to calculate the minimum distance of the grid (Fig 3B). The source point is a point on the source object that is used to calculate the minimum distance from the center of the grid (Fig 3C). The source-point information includes the coordinates of the point. These matrices comprise the geographical distance field. The geographical distance field quantifies the region of influence of each point of the spatial objects and establishes the corresponding relationship between each point of the spatial objects and the pixels of each grid. Therefore, feature points that the regions of influence reach or that are beyond the buffer boundary can be identified by using the source point tracking of buffer-boundary pixels.

Line simplification that is based on the geometric characteristics of bends can effectively preserve the shape characteristics [21]. Therefore, this article identifies the feature points that the regions of influence reach or that are beyond the buffer boundary, and then bends are selected by using these feature points. Then, these bends are classified by using the geometrical properties of the bends. Finally, we use the geometric characteristics of these bends and arcs to replace the deleted bends and simplify the coastline. Using arcs to replace the deleted bends can maintain the concavity and convexity of the coastline and avoid changing the deleted portions from bends to straight lines.

#### Detecting bends and recognizing the coastline vertices that the regions of influence reach or that are beyond the buffer boundaries based on the geographical distance fields

**1**. **Generation of geographical distance fields**

Geographical distance fields were created through Hu’s geographical distance transformation, which iteratively updates the distances by using two scanning propagations [43].

Geographical-distance transformation first establishes the relationship between the Earth's surface and raster space through a map projection. This article uses the equidistant cylindrical projection to establish this relationship. The calculation of equidistant cylindrical projection is based on the formula in the textbook authored by Snyder[44].

For example, when the Earth's surface satisfies λ_*min*_≤λ≤λ_*max*_ and *φ*_*min*_≤φ≤φ_*max*_, (λ, φ) are the latitude and longitude coordinates. We can use the forward formulas of equidistant cylindrical projection to generate a cylindrical equidistant map that satisfies x_*min*_≤x≤x_*max*_ and y_*min*_≤y≤y_*max*_, where (x, y) are the Cartesian coordinates. Next, the cylindrical equidistant map is divided into an m*n raster space according to the specified grid resolution, where m is the number of rows and n is the number of columns. Therefore, each point on the cylindrical equidistant map falls into a pixel (i, j), where i is the row index and j is the column index. When the centroid coordinates of the lower-left pixel are (x_min_, y_min_), the centroid coordinates of pixel (i, j) can be calculated as follows:

$$x_{c}=x_{min}+i*r,y_{c}=y+j*r$$ (1)

where (x_**c**_, y_c_) are the centroid coordinates of pixel (i, j) on the cylindrical equidistant map.

The latitude and longitude of the centroid of each pixel can also be calculated by the inverse formulas of equidistant cylindrical projection.

When establishing this relationship, the centroids of the foreground grids (which are the grids that the object of the distance transform falls into) are the points that are on the object of the distance transform. Three scenarios exist when calculating the foreground coordinate values:

If one vertex of the object of the distance transform falls into the foreground grid, the centroid of the foreground pixel is this vertex;

If multiple vertices of the object of the distance transform fall into the foreground grid, the centroid of the foreground pixel is the nearest vertex to the point that is calculated by Eq (1);

If no vertex of the object of the distance transform falls into the foreground grid, the centroid of the foreground pixel is the midpoint of the line segment that is captured by the foreground grid.

The implementation processes of the geographic distance transform are provided below:

Create an image that consists of the coastline as the foreground and other objects as the background. Then, the initial distance value (D_ij_) is assigned to the pixel (i, j) to initialize the distance map. The distance value (D_ij_) of the foreground pixels is assigned to 0, and the distance value (D_ij_) of the background pixels is assigned a sufficiently large value K.

**Fig 4:**
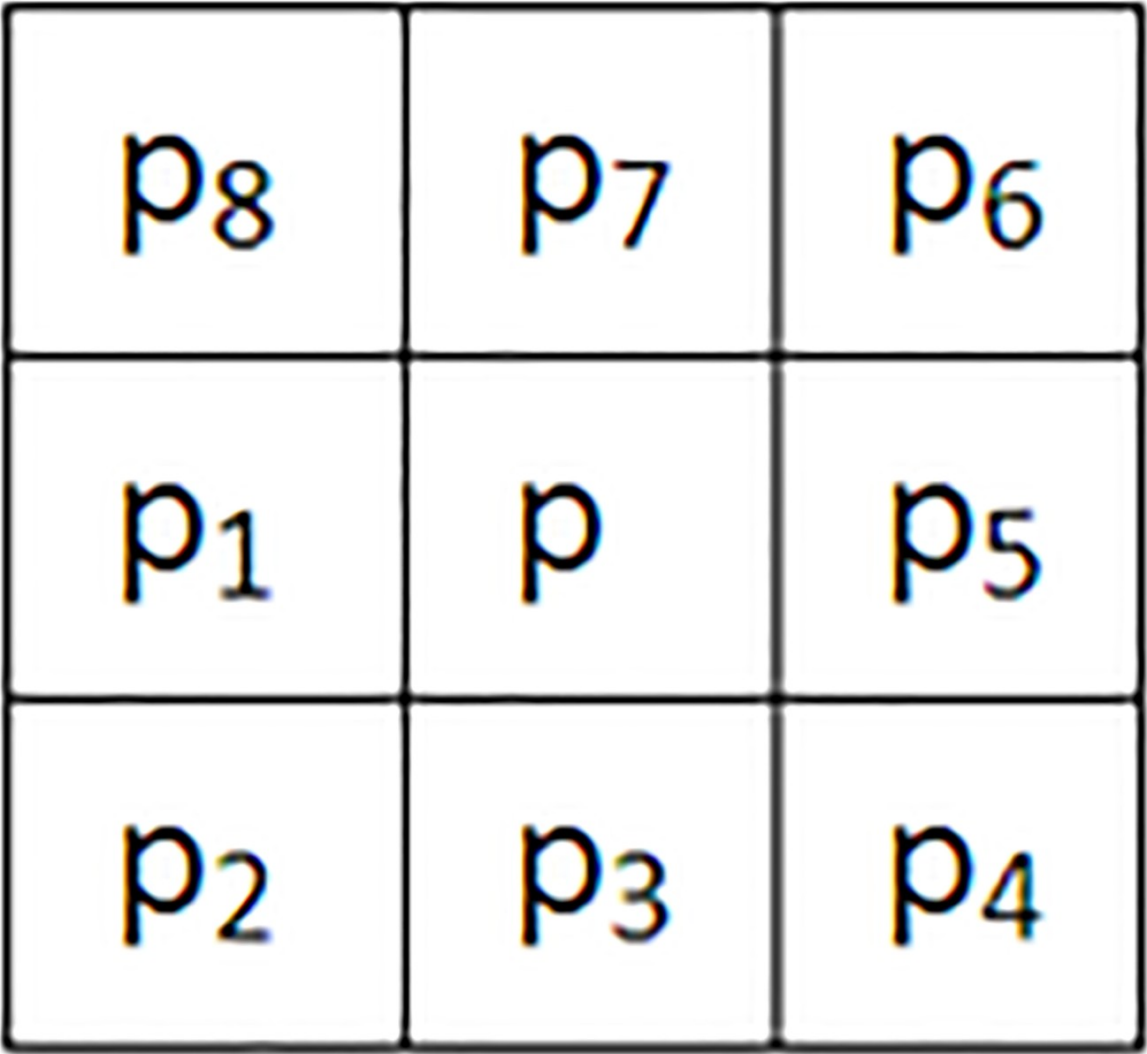
The neighborhood template of a cell.

Forward scan the image, update the distance value of each pixel by using Fig 4 and Eq (2), and record the minimum-distance source object and the source-point coordinate values:

$$D_{ij}=min\{D_{ij},N_{ij}\left(g_{1}\right),N_{ij}\left(g\right),N_{ij}\left(g_{3}\right),N_{ij}\left(g_{4}\right)\}$$ (2)

Reverse scan the image, and update the distance value, the minimum-distance source object and the source-point coordinate values of each pixel by using Fig 4 and Eq (3):

$$D_{ij}=min\{D_{ij},N_{ij}\left(g_{5}\right),N_{ij}\left(g_{6}\right),N_{ij}\left(g_{7}\right),N_{ij}\left(g_{8}\right)\}$$ (3)

*N*_*ij*_(*g*), where g = g1 … g8 is the distance between pixel (i, j) and the nearest foreground pixel of its neighbor “g” during the iteration period. By calculating *N*_*ij*_(*g*), the pixel’s central coordinate P (I, J) in Cartesian coordinates is mapped to latitude-longitude coordinates by Eq (1). Then, the distance is calculated by using Vincente's inverse distance formula.

Therefore, no positional errors in the source points of the geographical distance field are generated by the above approaches.

Coastline generalization has the principle of land priority, so we use land as the foreground and the ocean as the background during the geographical-distance transformation.

**2**. **Recognition of coastline vertices that influence the buffer consistency from the original coastline to the generalized coastline**

Generally, the generation of a buffer based on the distance field involves the following steps. First, create a binary image based on the distance field according to the buffer radius. Then, track and match the binary image’s boundary to generate the buffer-boundary pixel sequence. Finally, use the information regarding the buffer-boundary pixel sequence to determine the buffer boundary.

This process shows that the source points of the binary image’s boundary pixels are coastline points that influence the generation of the buffer boundary. Therefore, the SPTBB can be used to identify the coastline vertices that influence the buffer consistency from the original coastline to the generalized coastline.

**Fig 5:**
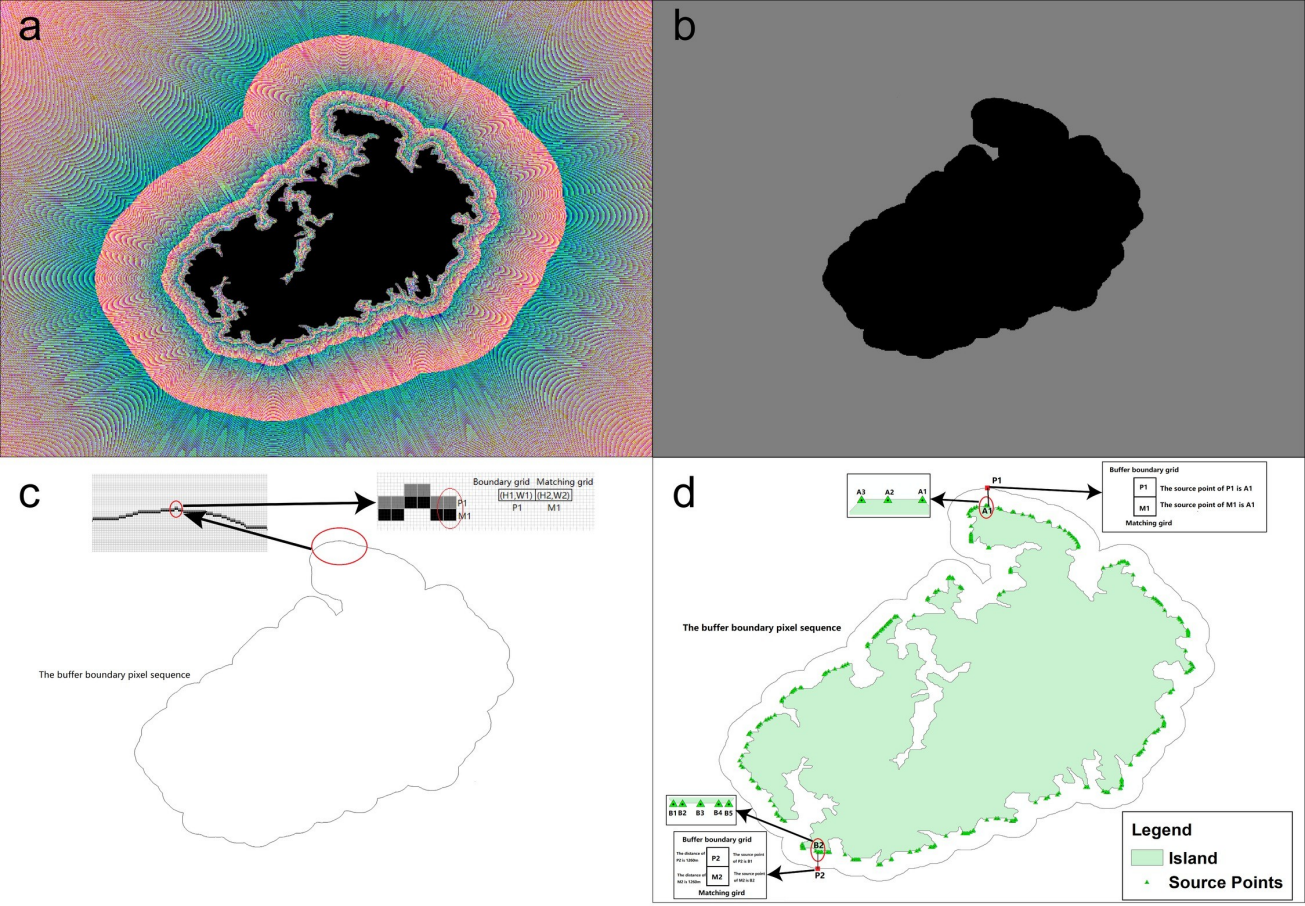
Source point tracking of buffer boundaries. (a) Geographical distance field from coastline-boundary tracking and matching; (b) binary image based on the raster matrix of the spatial distances according to the buffer radius (1250 m); (c) buffer-boundary pixel sequence that was generated by the tracking and matching of the buffer boundaries; (d) source points of the buffer boundary that were determined by traversing the buffer-boundary pixel sequence.

The SPTBB is conducted via the following steps. First, generate the geographical distance field by using geographical-distance transformation (Fig 5A). Then, create a binary image based on the raster matrix of the spatial distances according to the buffer radius (Fig 5B). Next, track and match the buffer boundary to generate the buffer-boundary pixel sequence (Fig 5C). Finally, traverse the buffer-boundary pixel sequence to determine the corresponding source point of each boundary pixel based on the raster matrix of the source coordinates (Fig 5D).

When tracking and matching the buffer boundaries, we first find a boundary grid of the binary image as the starting point of the search and then use the template in Fig 4 to search in a clockwise direction. The boundary grids are matched by using the four neighborhoods of the template in Fig 4 (i.e., upper, right, lower, and left). After tracking and matching the boundaries, the adjacency matching relationships of all the buffer boundary grids can be determined and a continuous buffer-boundary grid-pair sequence is obtained (Fig 5C).

To determine the corresponding source point of each boundary pixel, we first determine whether the source points of the boundary grid and the matching grid are the same. If so, the source point is the identified point. If the points are different, the source point of the grid with a more similar distance to the buffer radius is taken as the identified point (Fig 5D).

**Fig 6:**
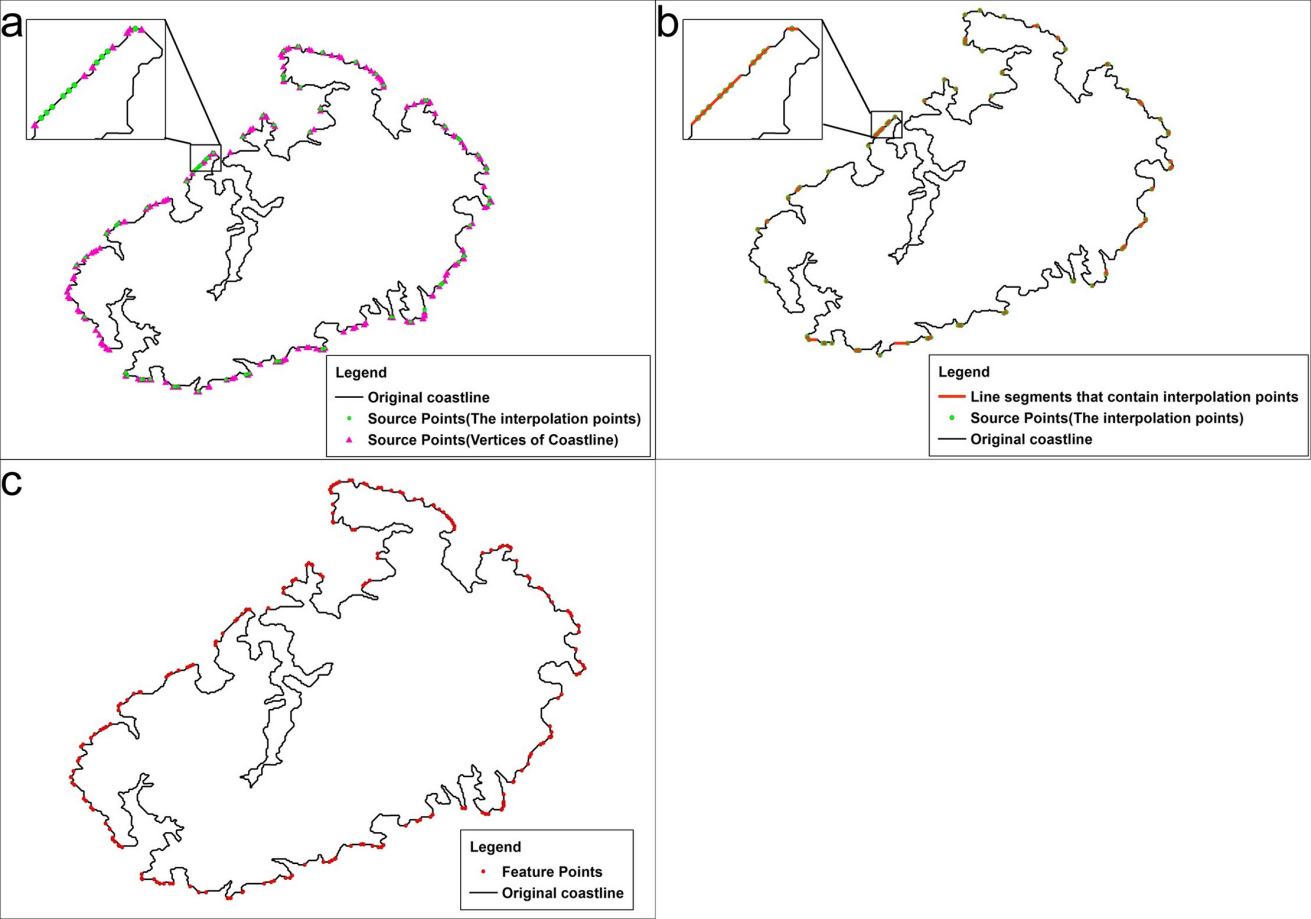
Identification of the key points that influence the generation of buffer boundaries. (a) Composition of the source points; (b) line segments that contain interpolation points; (c) feature points that influence the buffer-boundary generation.

The points that are recognized by the SPTBB contain some points that are not vertices of the coastline (Fig 6A). These points are called interpolation points, which are generated during coastline rasterization. During this process, the midpoint of the line segment that is captured by the foreground grid is used as the centroid of the foreground pixel when no coastline vertex falls into the foreground grid. Essentially, the line segments that contain these points are also features that influence the buffer-boundary generation (Fig 6B); that is, the vertices of these segments are also feature points.

Therefore, the feature points of the coastline that influence the buffer-boundary generation consist of the coastline vertices that are identified by the SPTBB and the vertices of the line segment that contain the interpolation points (Fig 6C).

In this article's method, the radius of the buffer can be determined by using the back-and-forth buffering principle; that is, the buffer radius equals one half the generalization tolerance.

**3**. **Recognition of bends**

**Fig 7:**
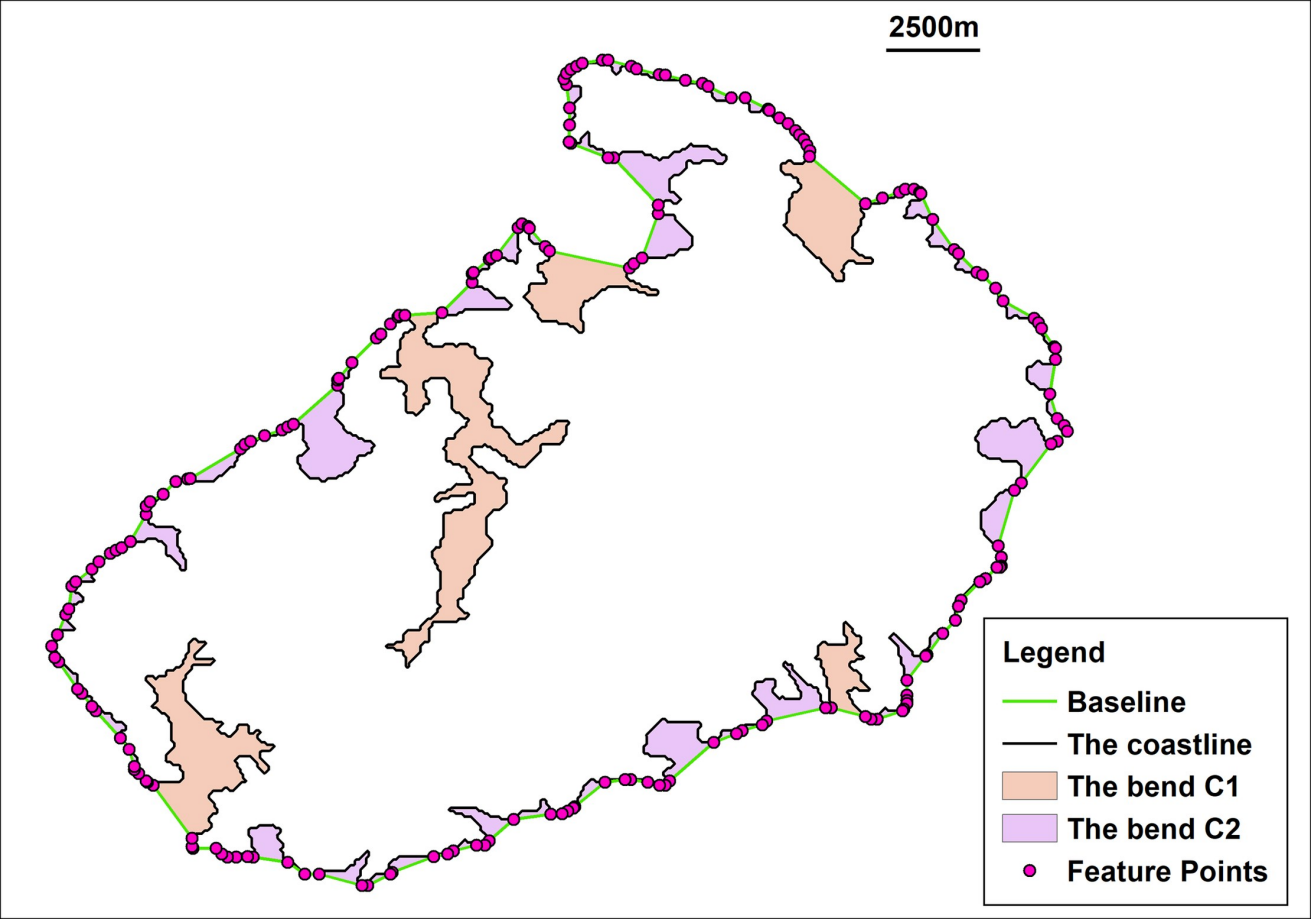
Identification and classification of bends. This figure shows the bends that were recognized by the SPTBB (the width of the buffer was 1250 m) and the classification results of the bends based on the generalization tolerance (2500 m).

After the feature points of the coastline that influence the buffer-boundary generation are identified, these points are used to generate a baseline, and the bends that do not contain the feature points can be recognized (Fig 7). These bends are the objects of the coastline simplification

#### Classification of bends

We first classify the bends before they are simplified to preserve the shape characteristics of the simplified coastline as much as possible. The width and length of the bend are used as criteria.

The width of a bend can be calculated by using Eq (4):

$$Width_{bend}=Dis(StartPt,EndPt)$$ (4)

where *Width*_*bend*_ is the width of the bend, *Dis* (*StartPt*,*EndPt*) is the distance from the starting vertex of the bend to the ending vertex, and *StartPt* and *EndPt* represent the starting and ending vertices of the bend, respectively.

The length of a bend can be calculated by using Eq (5):

$$Length_{bend}=Area_{bend}/Width_{bend}$$ (5)

where *Length*_*bend*_ is the length of the bend, *Width*_*bend*_ is the width of the bend, and *Area*_*bend*_ is the area of the bend.

Bends are classified into the two categories C1 and C2:

Class C1: the length or width of the bend is greater than the tolerance of the generalization (Fig 7).

Class C2: neither the length nor the width of the bend is greater than the tolerance of the generalization (Fig 7).

#### Bend simplification and the treatment of concave and convex maintenance

**1**. **Bend simplification**

According to the length and width of bends, which are as reference indicators for coastline simplification, class C1 cannot be deleted. However, class C1 includes small bends and thus should be further simplified. In this step, we use the Delaunay triangulation method [1] to detect bends and establish a hierarchical tree. The bends in this hierarchical tree that have a medial axis length that is no greater than the generalization tolerance are then deleted. However, when the width of the bend is greater than the generalization tolerance, the medial axis length of the bends in the hierarchical tree are less than the generalization tolerance; this type of bend only retains the first level of bends in the hierarchical tree. Through the above process, the simplified bends both retain the overall morphological characteristics of the original bends and eliminate the detailed features of these bends.

Although class C2 can be deleted, the concavity and convexity of the coastline will change, which will directly affect the results of the buffer analysis. The generalized coastlines should maintain their concavity and convexity after any C2 bends are deleted to reduce the effect of changes in the coastline morphology on the buffer analysis’ accuracy.

****2. **Treatment of the concave and convex maintenance of the coastline**

**Fig 8:**
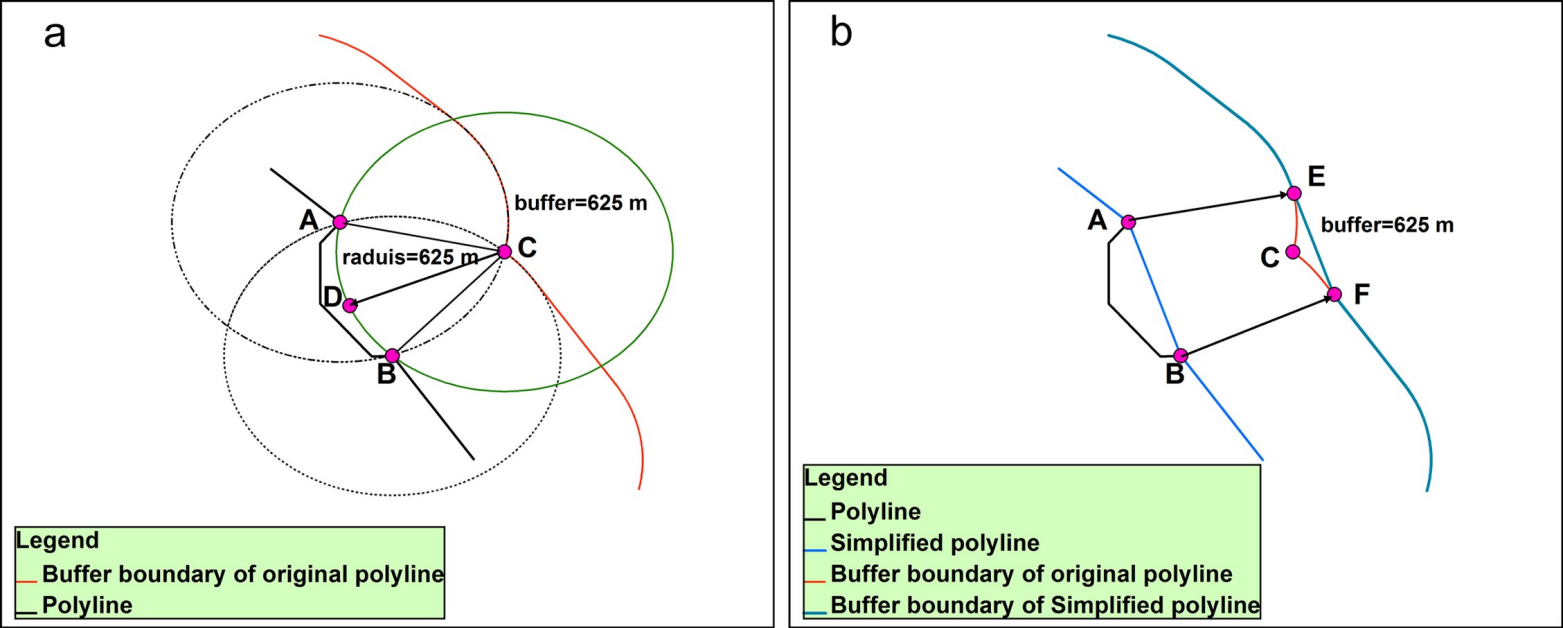
Processes that ensure buffer consistency. (a) Arc creation and (b) buffer creation after the coastline is simplified by direct deletion. $\overset{◠}{\mathrm{AB}}$ extends from point A to point B through point D on circle C. Circle C is centered at point C, and the radius of the buffer is the radius of circle C. Point C is the point of intersection of Circle A and Circle B, which are centered at point A and point B, respectively, and the radius of the buffer is their radius. Point C is the point of transition on the buffer boundary where the original point transfers from A to B.

In contrast to how changing from bends to straight lines deletes certain components, using arcs with radii that equal the width of the buffer to replace the deleted C2 bends can reduce changes in the concavity and convexity of the coastline, preserve the morphological characteristics of the coastline as much as possible, and reduce the effect of changes in the coastline morphology on the buffer analysis’ accuracy. The creation of arcs is shown in Fig 8, and this process is accomplished in vector space.

### Setting important parameters

The geographic distance field that is used in this paper is based on a discrete grid structure, and its accuracy is affected by the grid resolution. When the grid resolution is large, the rasterization process will cause a large number of features on the coastline to be lost, and these lost features will not be involved when generating the distance field. When this distance field is used for the SPTBB, a large amount of important information will be lost. When the grid resolution is small, the accuracy of the distance field will be better, but the computational requirements will also increase. Setting a reasonable grid resolution can effectively balance the accuracy and computational efficiency.

#### Setting the grid resolution of the geographical distance field

To effectively balance the accuracy and computation efficiency of distance transformation, the grid resolution can be calculated using Eq (6):

R=C*S (6)

where R is the grid resolution, C is a constant equal to 0.02cm which represents the minimum gap (0.02 cm) between two lines that can be distinguished on a map, and S is the scale denominator of the coastline.

The coastline is generally represented by a polyline with the weight of 0.15–0.25 mm in cartography[45]. Two lines on a map can be distinguished when they are separated by at least 0.2 mm [16]. In addition, to meet the needs of visualization, the width of the smallest feature of the coastline at different scales should be greater than this distance of 0.2 mm on the map. Therefore, the grid resolution of the distance transformation is set with reference to the calculated value from Eq (6), which ensures that each feature on the coastline will participate in the generation of the geographic distance field during the distance transformation.

**Fig 9:**
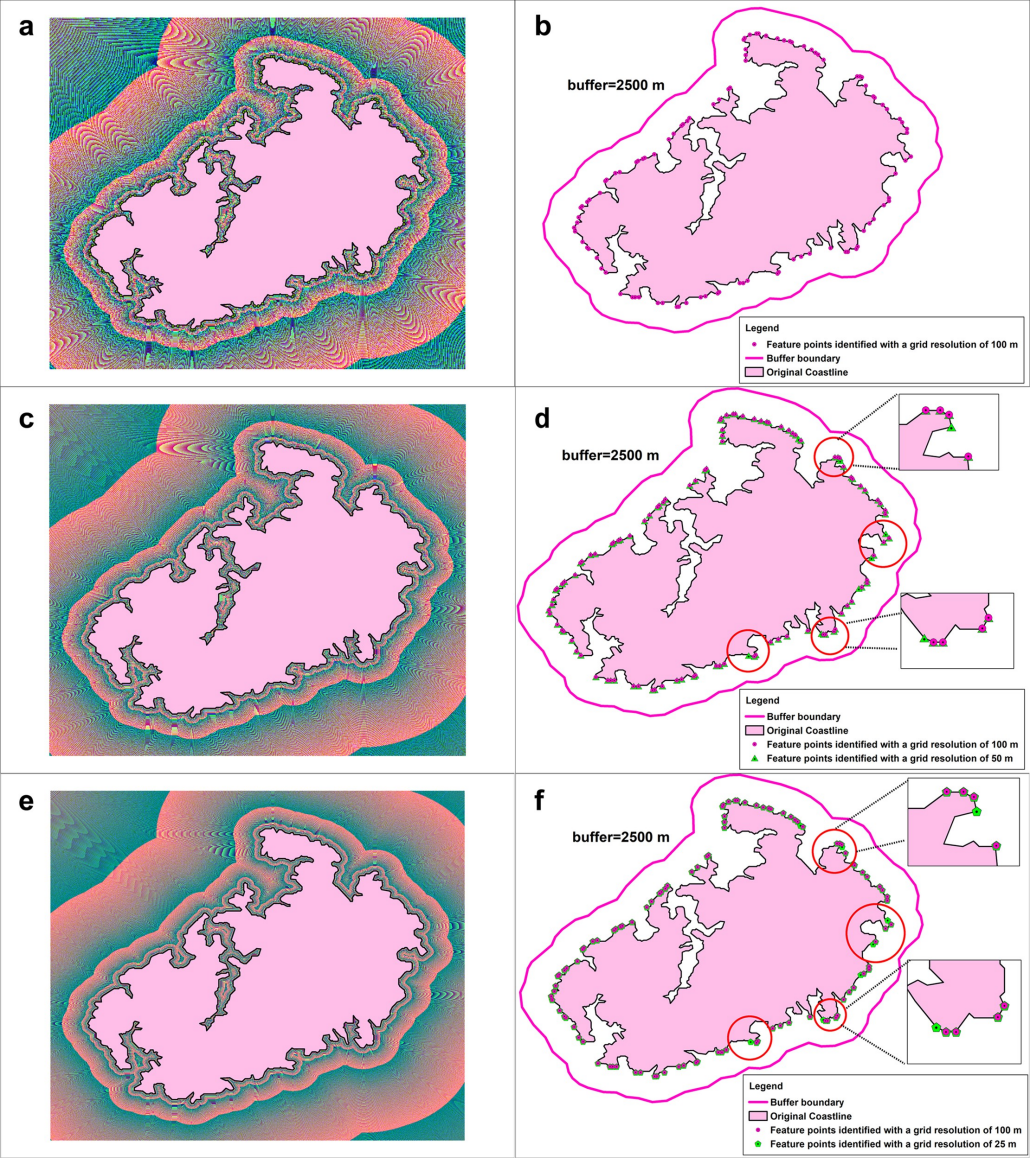
Distance field of a coastline (1:250 K) with a grid resolution of 100 m, 50m, and 25 m and the identified vertices of the coastline that the regions of influence reach or that are beyond the buffer boundary based on the geographical distance fields with different grid resolutions. (a)-(b) Distance field with a grid resolution of 100 m and the identified feature point; (c)–(d) distance field with a grid resolution of 50 m and the identified feature point; (e)–(f) distance field with a grid resolution of 25 m and the identified feature point.

For example, when the scale of coastline is 1:250K, the grid resolution calculated by Eq (6) is 50 meters. Fig 9 shows the distance field of the coastline (1:250 K) with a grid resolution of 100 m, 50 m, and 25 m and the identified vertices of the coastline that the regions of influence reach or that are beyond the buffer boundary based on the geographical distance fields with different grid resolutions.

As shown in Fig 9, the feature points that are identified in the different geographical distance fields are the same when the grid resolution is less than or equal to the calculated value from Eq (6). Therefore, the grid resolution from Eq (6) can balance the accuracy of the feature-point recognition and the computational efficiency.

Next, we will introduce the assessment of the original and generalized coastlines.

### Assessment of the buffer consistency from the original coastline to the generalized coastline

#### Definition of the buffer-consistency coefficient

To more comprehensively describe the error of the buffer, the buffer consistency coefficient (BCC_*λ*_) is used as a description and evaluation index for the buffer consistency from the original coastline to the generalized coastline. The BCC_*λ*_ is defined in Eq (9):

$${\mathrm{BCC}}_{\lambda }=\Delta \mathrm{Area}/\left(l*\lambda \right)*100\%$$ (7)

where BCC_λ_ is the buffer consistency coefficient, ΔArea is the area of the polygon that consists of the buffer boundaries from the generalized and original coastlines, *l* is the length of the buffer boundary of the original coastline, and λ is the buffer radius. If the two curves are the same, the area of the polygon that consists of these curves equals zero. The overall offset distance from the buffer of the generalized coastline to the buffer of the original coastline can be calculated by dividing ΔArea by *l*. Finally, the ratio of the overall offset distance to the buffer radius can be calculated from the overall offset distance divided by λ. Therefore, BCC_λ_ can be used to evaluate the error of the buffer.

## Experimental results

### Experimental setting

A generalization experiment of the coastline of Stewart Island (scale 1:250 K) was performed to demonstrate the viability of the SPTBB method. The data that were used were retrieved from the National Oceanic and Atmospheric Administration (NOAA). A comparative analysis was completed by using the method of Wang and Müller (WM; 1998), which is available in ArcGIS10.0.

The reasons for choosing the WM method for the comparison are as follows:

1. The WM method typically produces results that are more representative of the original coastline and are more aesthetically pleasing.
2. Similar to our method, the WM method is based on bend detection. Therefore, a comparison with the WM method is directly applicable.

### Results

**Fig 10:**
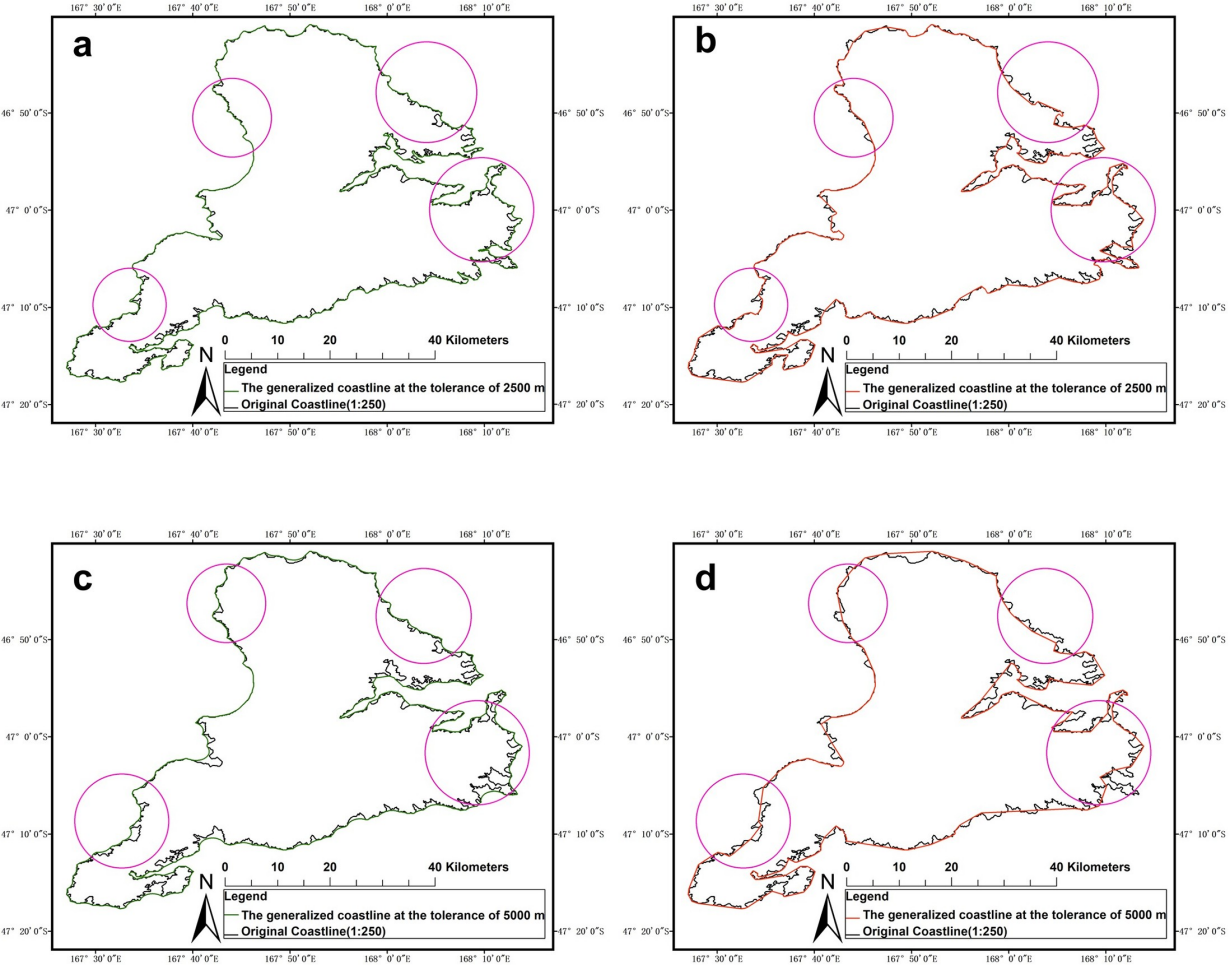
Coastlines of Stewart Island (1:250 K) that were generalized by the SPTBB and WM methods. (a) and (b) Results from the SPTBB method; (b) and (d) results from the WM method. Circle show the locations where capes on the coastline were deleted.

Fig 10 shows the coastlines of Stewart Island (1:250 K) that were generalized by using the SPTBB method and the WM method. Fig 10A and 10B show the coastlines of Stewart Island at 2500 m and 5000 m that were generated by the SPTBB method. Fig 10C and 10D show the coastlines of Stewart Island at 2500 m and 5000 m that were generated by the WM method. Fig 10 shows that the coastlines that were generalized by these two methods were generally similar. The smaller the tolerance of the generalization, the more similar the generalized coastlines became. Although the coastlines that were generalized by these two methods were generally similar, the circles in Fig 10 show that the SPTBB method filled in concave areas along the coastline, removed small bays, preserved capes, and maintained the concavity and convexity of the generalized coastline. This result overcame one problem of the WM method, in which key convex shapes along the coastline were not preserved.

**Fig 11:**
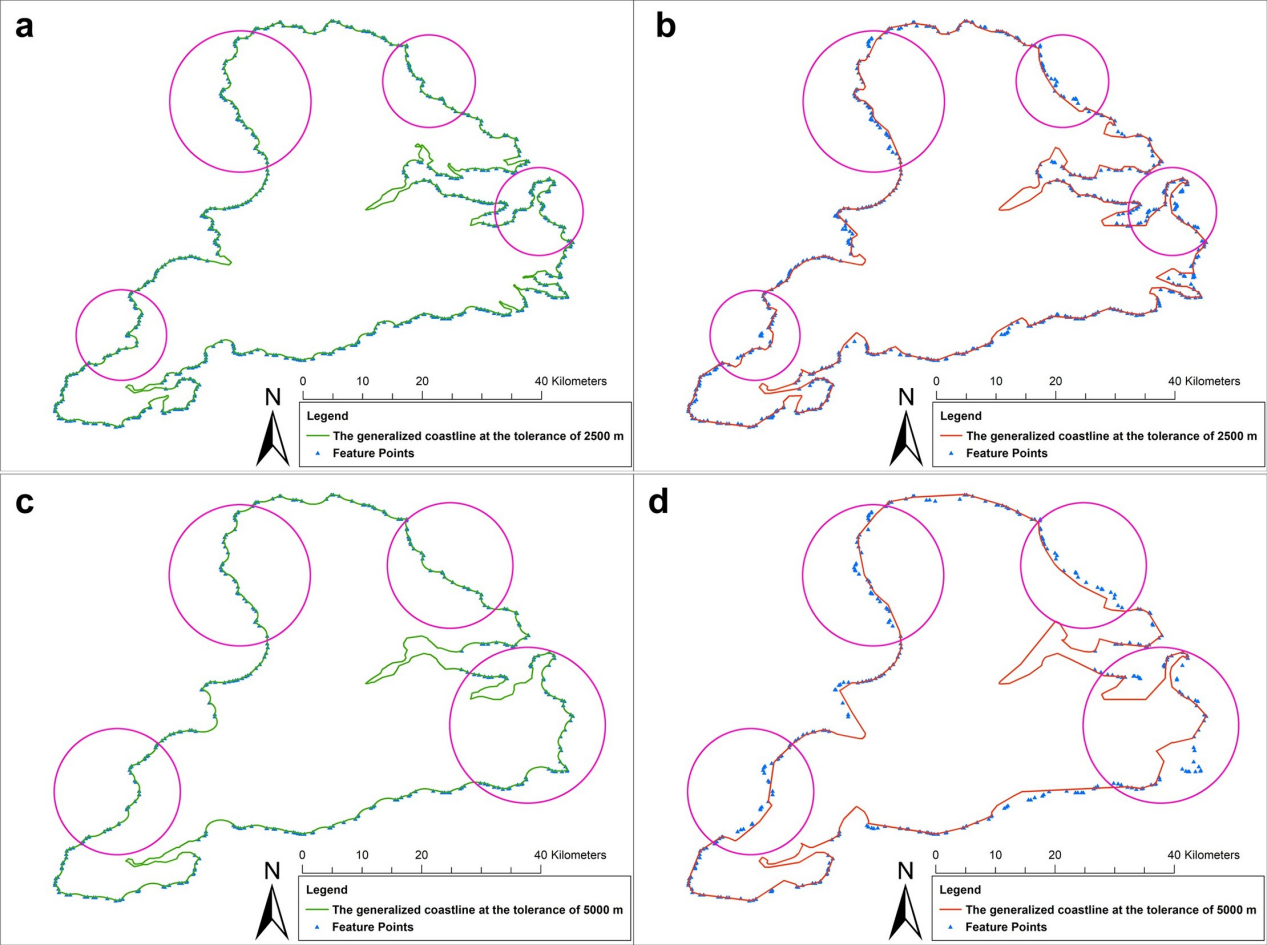
Results of the overlay comparison between the generalized coastlines and feature points that affected buffer-boundary generation. (a) and (c) Results of the overlay comparison between the generalized coastlines and feature points that affected buffer-boundary generation (the radius of the buffer was 1250 m); (b) and (d) results of the overlay comparison between the generalized coastlines and feature points that affected buffer-boundary generation (the radius of the buffer was 2500 m). Circle show the locations where feature points on the coastline were deleted.

Fig 11 shows the results of the overlay comparison between the generalized coastlines and feature points that affected buffer-boundary generation. Fig 11A and 11C show the results of the overlay comparison between the coastlines that were generalized by the SPTBB method and feature points that affected buffer-boundary generation. Fig 11B and 11D show the results of the overlay comparison between the coastlines that were generalized by the WM method and feature points that affected buffer-boundary generation. Fig 11 indicates that the SPTBB method was more effective than the WM method in retaining the feature points of the coastline that influenced the buffer-boundary generation.

**Fig 12:**
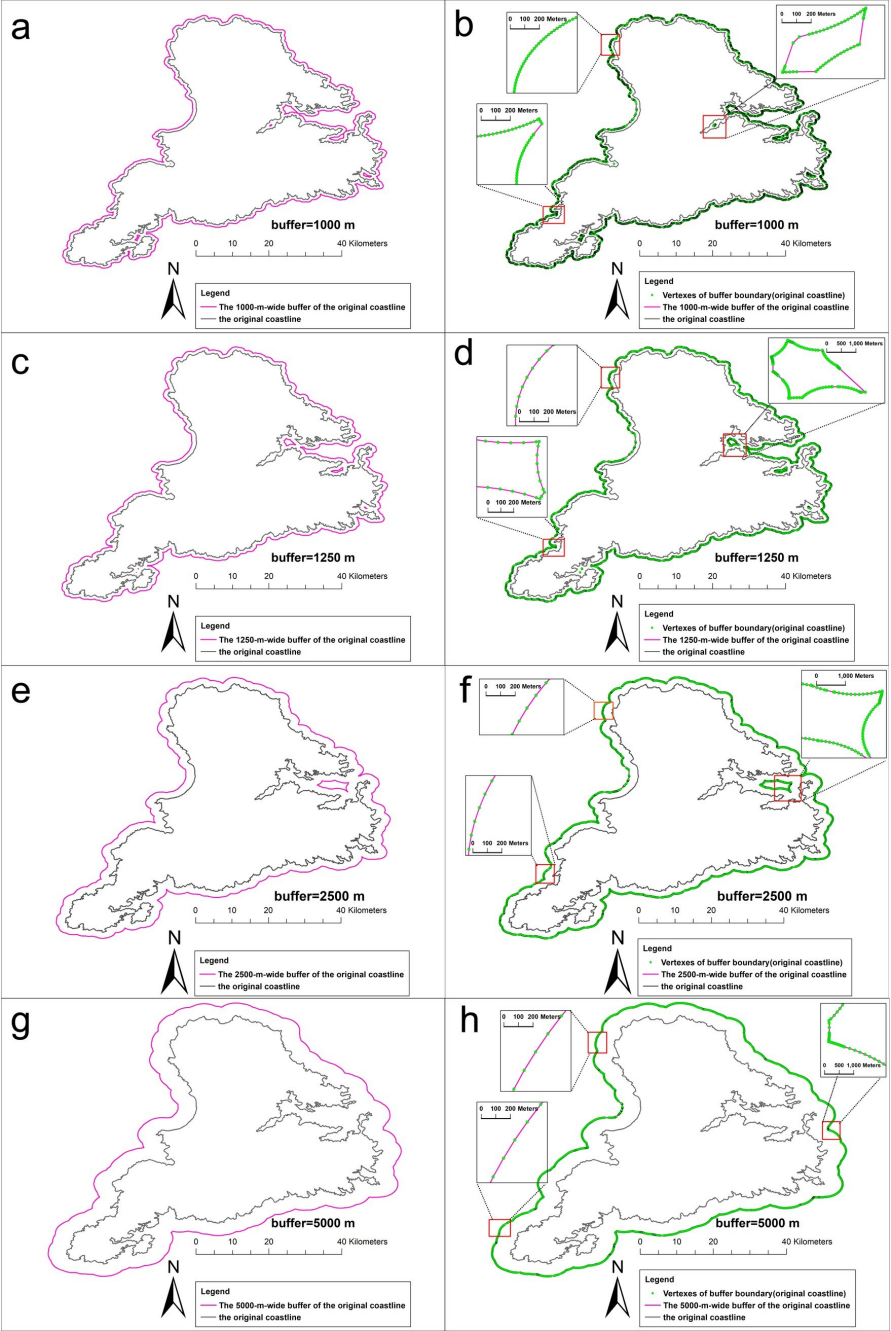
The results of the buffer analysis of the Stewart Island coastline (1:250K). (a) A 1000-m-wide buffer created for the Stewart Island coastline (1:250 K); (b) the vertexes on the buffer boundary (the radius of the buffer was 1000 m); (c) A 1250-m-wide buffer created for the Stewart Island coastline (1:250 K); (f) the vertexes on the buffer boundary (the radius of the buffer was 1250 m). (e) A 2500-m-wide buffer created for the Stewart Island coastline (1:250 K); (f) the vertexes on the buffer boundary (the radius of the buffer was 2500 m). (g) A 5000-m-wide buffer created for the Stewart Island coastline (1:250 K); (h) the vertexes on the buffer boundary (the radius of the buffer was 5000 m).

**Fig 13:**
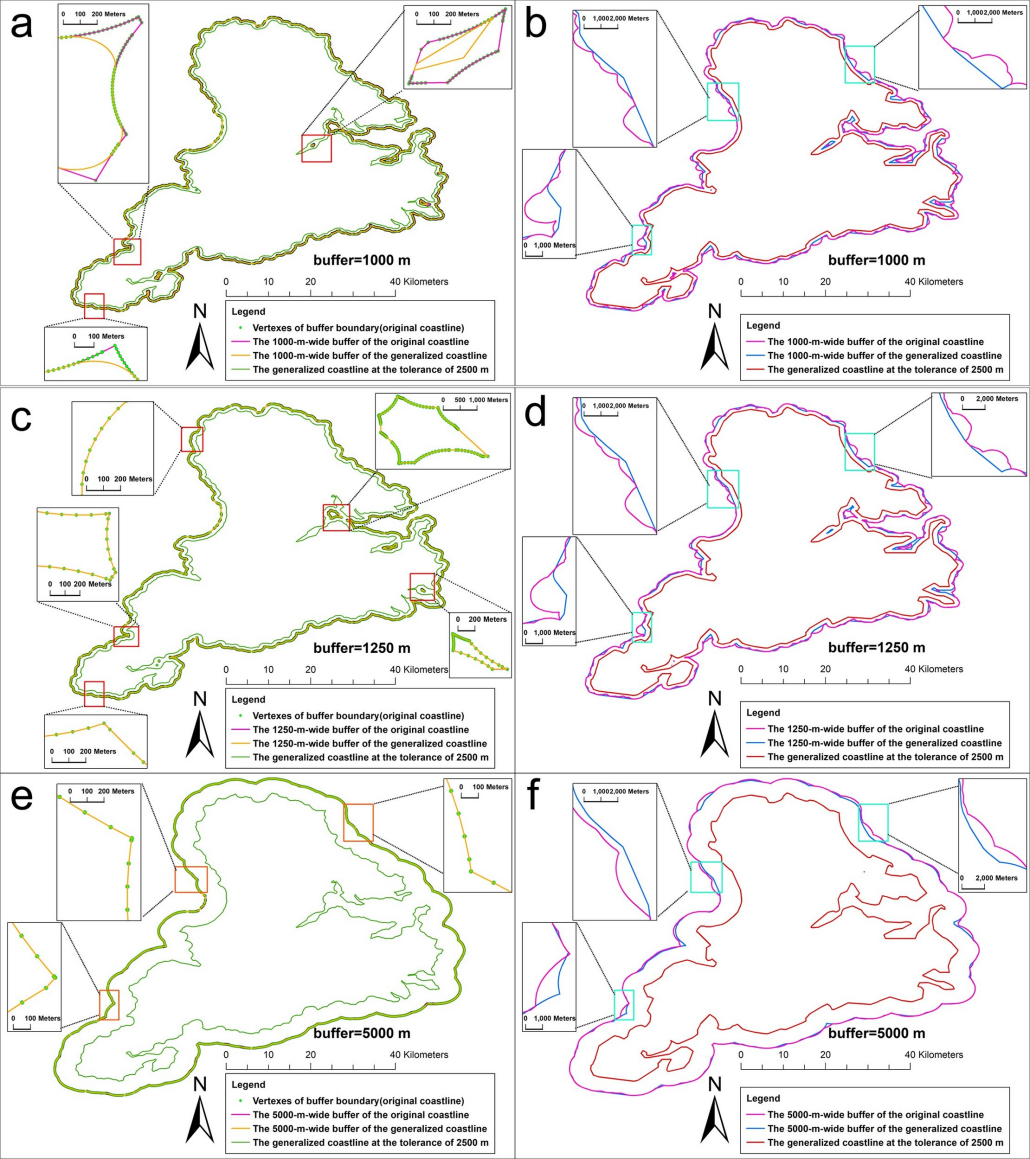
The results of the buffer analysis of the coastlines at 2500m generated by the SPTBB method and WM method. (a),(c) and (e) the results of the buffer analysis of the coastlines at 2500m generated by the SPTBB method; (b),(d) and (f) the results of the buffer analysis of the coastlines at 2500m generated by the WM method. Rectangle show comparison of the buffer boundary of the generalized coastline with the buffer boundary of the original coastline.

**Fig 14:**
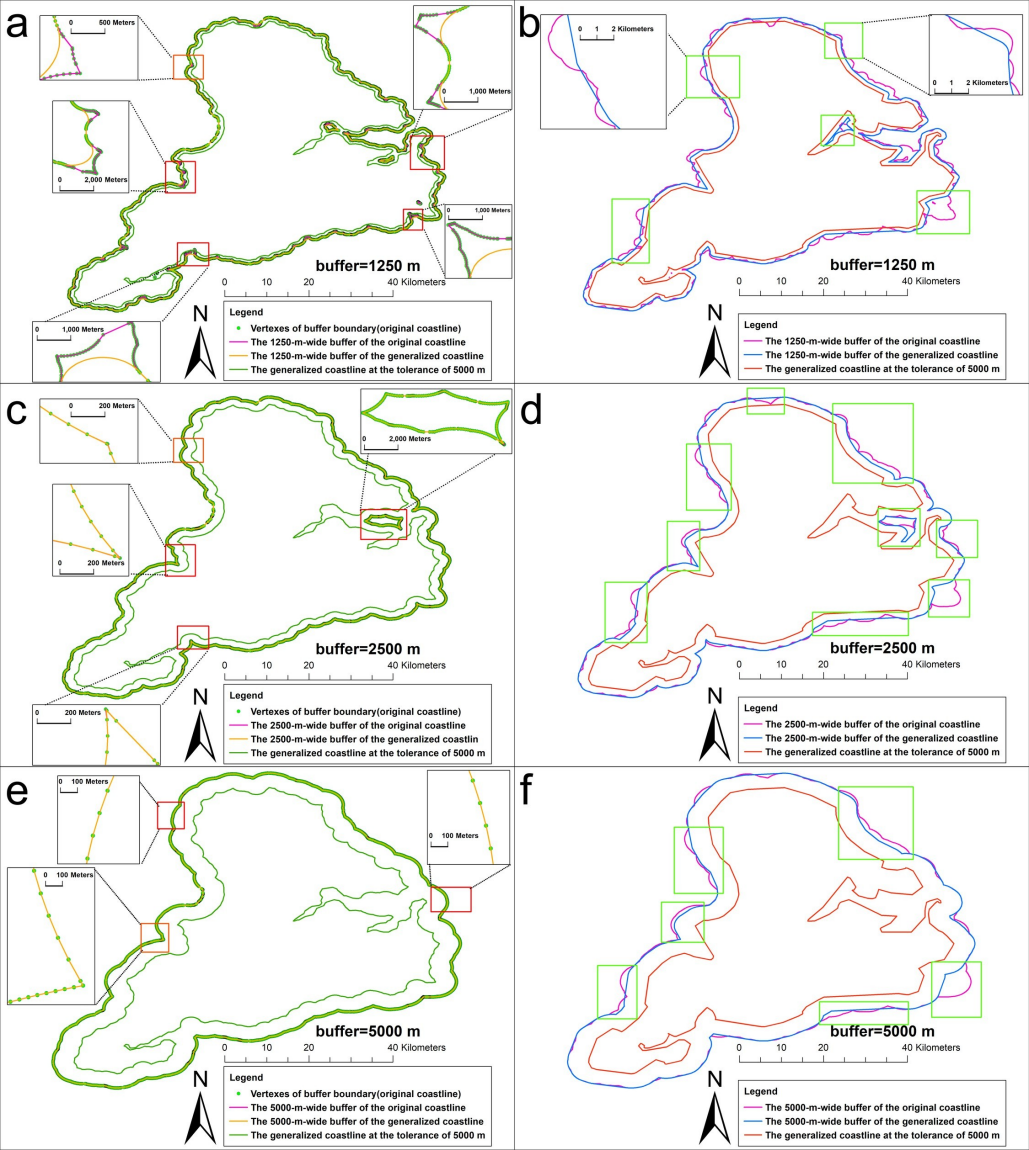
The results of the buffer analysis of the coastlines at 5000m generated by the SPTBB method and WM method. (a),(c) and (e) the results of the buffer analysis of the coastlines at 5000m generated by the SPTBB method, (b),(d) and (f) the results of the buffer analysis of the coastlines at 5000m generated by the WM method. Rectangle show comparison of the buffer boundary of the generalized coastline with the buffer boundary of the original coastline.

Fig 12 shows the results of the buffer analysis of the original coastline at a radius of 5 km, 2.5km,1.25 km, and 1 km. Fig 13 shows the results of the buffer analysis of the generalized coastlines. The generalized coastlines were the coastlines that were generalized by the SPTBB and WM methods at a threshold of 2500 m. Fig 14 shows the results of the buffer analysis of the generalized coastlines at a radius of 5 km, 2.5 km, and 1.25 km. The generalized coastlines were the coastlines that were generalized by the SPTBB and WM methods at a threshold of 5000 m.

According to Figs 12–14, the SPTBB method maintained the seaward buffer boundaries from the original coastline to the generalized coastline when the distance was greater than one half the tolerance of the coastline generalization. Inconsistencies existed in the buffer-analysis results when the radius of the buffer was smaller than this value. However, the results of the buffer analysis of the coastlines that were generalized by the WM method at any radius were not consistent with the results of the buffer analysis of the original coastlines.

Table 1 and Table 2 shows the change in the BCC_λ_ value of Stewart Island. When using the SPTBB method, the value of BCC_λ_ was less than 0.01%, and the deviation distance was less than 0.1 m when the distance was greater than one half the tolerance of the coastline generalization. Then, the value of BCC_λ_ was obviously larger than the above results when the distance was smaller than one half the tolerance of the coastline generalization. BCC_λ_ and the deviation distance from the WM method were much larger than those from our method at all scales. Overall, the buffers of the generalized coastline from the WM method exhibited large deviations.

**Table 1:** Values of BCC_λ_ calculated for the generalized coastline: the values of l are 493396.463 m, 458071.358 m, 359001.678, and the values of λ are equal to 1km,1.25km, 5km. The generalized coastlines were the coastlines that were generalized by the SPTBB and WM methods at a threshold of 2500 m.

Coastlines	Buffer radius(λ)/m	ΔArea /m^2^	Distance(Deviation)/m	BCC_λ_/%
SPTBB method	1000	1975979.31	4.005	0.400485
	1250	22204.61	0.048	0.003878
	5000	729.81	0.002	0.000041
WM method	1000	53102792.8	107.627	10.762702
	1250	54642517.67	119.288	9.543058
	5000	56821626.32	158.277	3.165535

**Table 2:** Values of BCC_λ_ calculated for the generalized coastline: the values of l are 458071.358 m, 390152.2 m, 359001.678, and the values of λ are equal to 1.25km, 2.5km, 5km. The generalized coastlines were the coastlines that were generalized by the SPTBB and WM methods at a threshold of 5000 m.

Coastlines	Buffer radius(λ)/m	ΔArea /m^2^	Distance(Deviation)/m	BCC_λ_/%
SPTBB method	1250	20674329.71	45.133	3.610674
	2500	18215.14	0.047	0.001867
	5000	323.06	0.001	0.000018
WM method	1250	116168157.3	253.603	20.288220
	2500	138140591.9	354.068	14.162739
	5000	153646714.8	427.983	8.559666

Table 3 and Table 4 shows the distances from the vertices on the buffer boundary of Stewart Island (1:250 K) to the buffer boundary of the generalized coastline. When using the SPTBB method, the error of the buffer-analysis result between the generalized coastline and the original coastline could be ignored relative to the buffer radius when the distance was greater than one half the tolerance of the coastline generalization. In contrast, the error of the result of the buffer analysis was obviously larger. The buffer boundaries for the coastlines that were generalized by the WM method considerably changed.

**Table 3:** Distances from the vertexes of the buffer boundary (original coastline) to the buffer boundary of the generalized coastlines. The generalized coastlines were the coastlines that were generalized by the SPTBB and WM methods at a threshold of 2500 m.

Coastlines	Buffer boundary	Distance/m		Standard Deviation	Vertex numbers
		Maximum distance	mean distance		
SPTBB method	1000 m	1944.619	7.104	44.148	13454
	1250 m	7.483	0.152	0.479	4748
	5000 m	1.057	0.005	0.061	2094
WM method	1000 m	2035.128	229.794	294.333	13454
	1250 m	4703.312	222.232	336.810	4748
	5000 m	1491.685	169.085	267.432	2094

**Table 4:** Distances from the vertexes of the buffer boundary (original coastline) to the buffer boundary of the generalized coastlines. The generalized coastlines were the coastlines that were generalized by the SPTBB and WM methods at a threshold of 5000 m.

Coastlines	Buffer boundary	Distance/m		Standard Deviation	Vertex numbers
		Maximum distance	mean distance		
SPTBB method	1250 m	4316.766	123.850	421.999	4748
	2500 m	21.202	0.170	0.717	3048
	5000 m	1.057	0.005	0.054	2094
WM method	1250 m	6509.658	527.950	827.955	4748
	2500 m	6509.269	499.275	853.998	3048
	5000 m	6449.228	493.808	880.738	2094

## Discussion

According to the above results, the SPTBB method effectively maintained the overall coastline shape and caps of coastlines, reduced the displacement and any convex and concave changes after deleting bends, and preserved the feature points that influenced the buffer-boundary generation (the buffer radius equaled one half the generalization tolerance). Thus, the SPTBB method provided specific differences in the buffer analysis of coastlines before and after generalization.

According to the principle of land priority that was used in this paper, land was used as the foreground and the ocean was used as the background to perform the distance transformation. The radius of the source point tracking of the buffer boundaries was determined by using the principle of back-and-forth buffering. That is, the feature points of the coastline that the seaward regions of influence reached or that were beyond one half the generalization tolerance could be identified. Then, we used these feature points to generate a baseline, and bends that did not contain the feature points could be recognized. Therefore, coastline simplification based on the geometric features of these bends could effectively retain the key convex points on the coastline, remove small bays, and preserve capes and feature points that the regions of influence reached or that were beyond one half the generalization tolerance. In addition, the method in this paper used arcs with radii that equaled the width of the buffer that was selected to replace any deleted C2 bends, reducing the effect of changes in the coastline morphology on the buffer analysis’ accuracy and ensuring that the spatial influence of the buffer analysis of the preserved feature points remained unchanged when the radius of the buffer analysis was greater than one half the generalization tolerance. Therefore, the method in this paper could determine the differences in the buffer-analysis results between the generalized coastline and original coastline.

Although the coastlines that were generalized by the WM method and SPTBB method were generally similar, the WM method is a shape-preserving method and does not consider the buffer consistency. The WM method uses the inflection-point method to identify bends and recursively deletes bends whose geometric features are smaller than the generalization tolerance to simplify the coastline. The WM method cannot recognize the feature points of coastlines that influence the buffer-boundary generation or overcome the displacement and the convex and concave changes that are caused by deleting bends. Therefore, the results of the buffer analysis between the generalized coastline and the original coastline exhibited large errors.

## Conclusion and outlook

This article presents a coastline generalization method that is based on SPTBB. This method uses the geographical distance field to identify the feature points and bends that influence the buffer consistency from the original coastline to the generalized coastline, and this process simplifies bends and maintains the concavity and convexity characteristics in the generalization. This method was compared to the WM method. The results indicated that the SPTBB method can preserve the shape characteristics of coastlines. In addition, this method has another important characteristic: it can provide a radius for the buffer analysis under which the buffer analysis result between the original coastline and the generalized coastline is consistent. That is, the seaward buffer boundaries of the generalized coastline and the original coastline are consistent when the distance is greater than the tolerance of the coastline generalization. Improving the precision of the analysis of the spatial influence is important when using small scales to perform highly accurate spatial analysis over large areas. This characteristic has important practical value; for example, this approach avoids the inconsistencies that are associated with marine boundaries that are calculated by using coastlines with different scales.

However, the distance field is based on a discrete grid structure, and the expression of spatial entities based on the gridded data structure is not continuous. The method that was proposed in this article cannot overcome the effects of the grid resolution. This issue will be addressed in future studies.

## Supporting information

S1 File Dataset used in the methodology and experiment. (ZIP)
