# Supplementary figures and images for: A coastline generalization method that considers buffer consistency
Source: PLoS One. 2018 Nov 1;13(11):e0206565. doi: 10.1371/journal.pone.0206565 (PMC6211722; doi:10.1371/journal.pone.0206565)

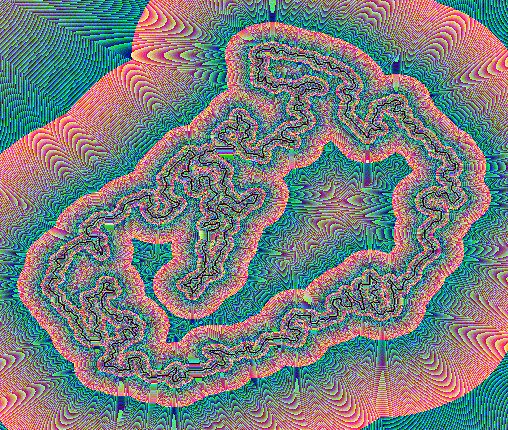

Supplement: S1 File — (ZIP) [file pone.0206565.s001.zip › Data used in methodology/the data used Fig 9/Dis-100.bmp]

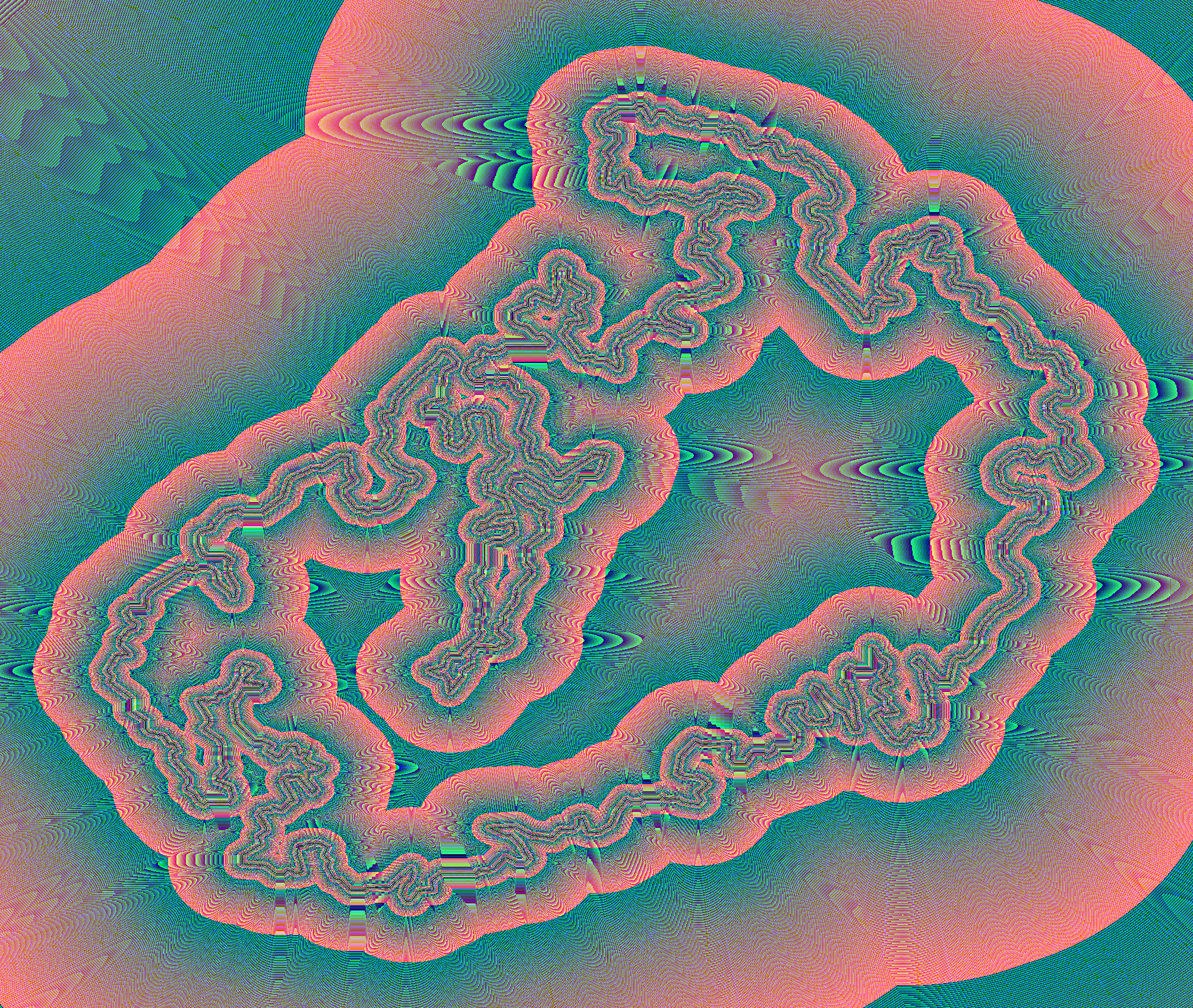

Supplement: S1 File — (ZIP) [file pone.0206565.s001.zip › Data used in methodology/the data used Fig 9/Dis-25.bmp]

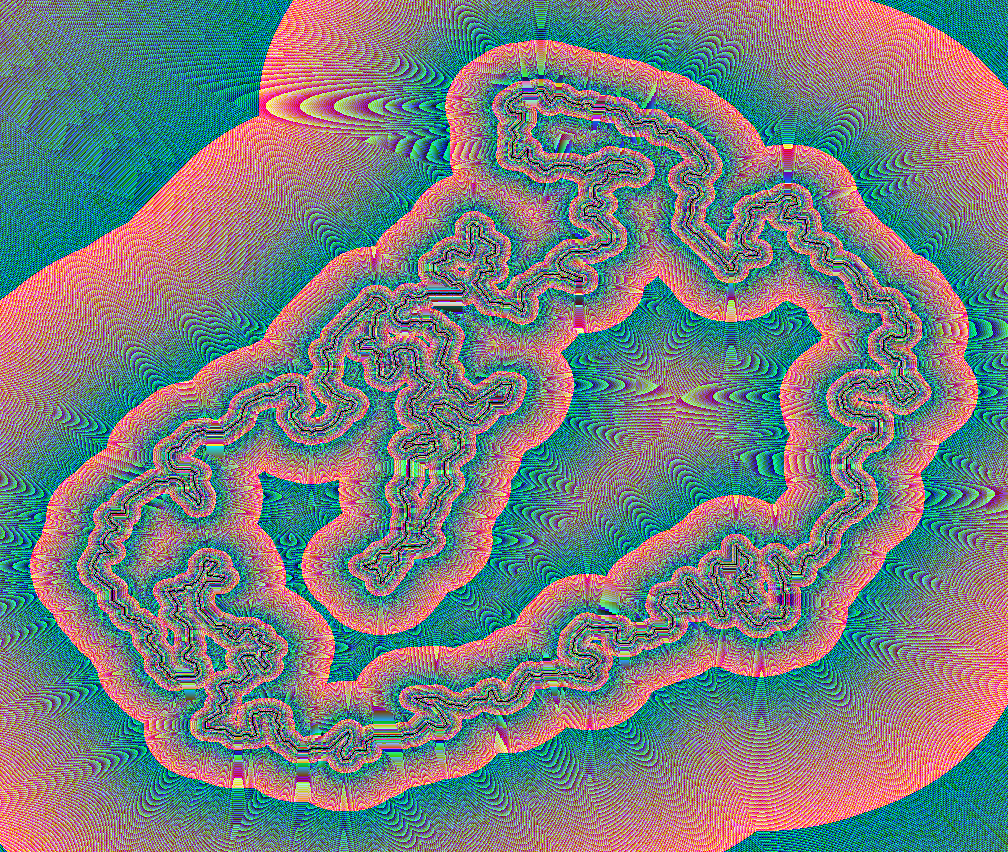

Supplement: S1 File — (ZIP) [file pone.0206565.s001.zip › Data used in methodology/the data used Fig 9/Dis-50.bmp]

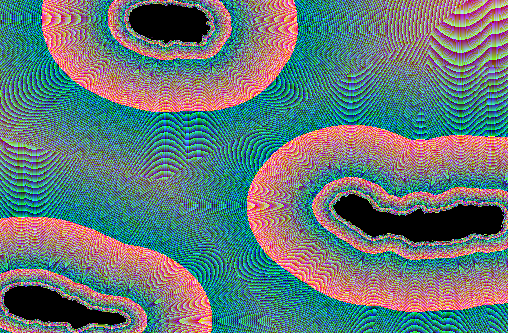

Supplement: S1 File — (ZIP) [file pone.0206565.s001.zip › Data used in methodology/the data used Fig 3/distance fileds/Dis.bmp]

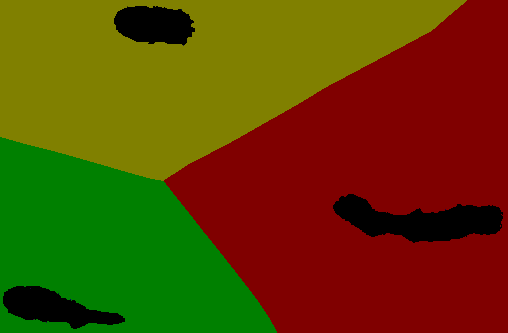

Supplement: S1 File — (ZIP) [file pone.0206565.s001.zip › Data used in methodology/the data used Fig 3/distance fileds/Ocp.bmp]

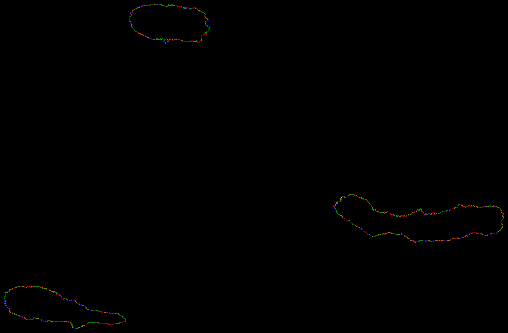

Supplement: S1 File — (ZIP) [file pone.0206565.s001.zip › Data used in methodology/the data used Fig 3/distance fileds/xxxxx.bmp]

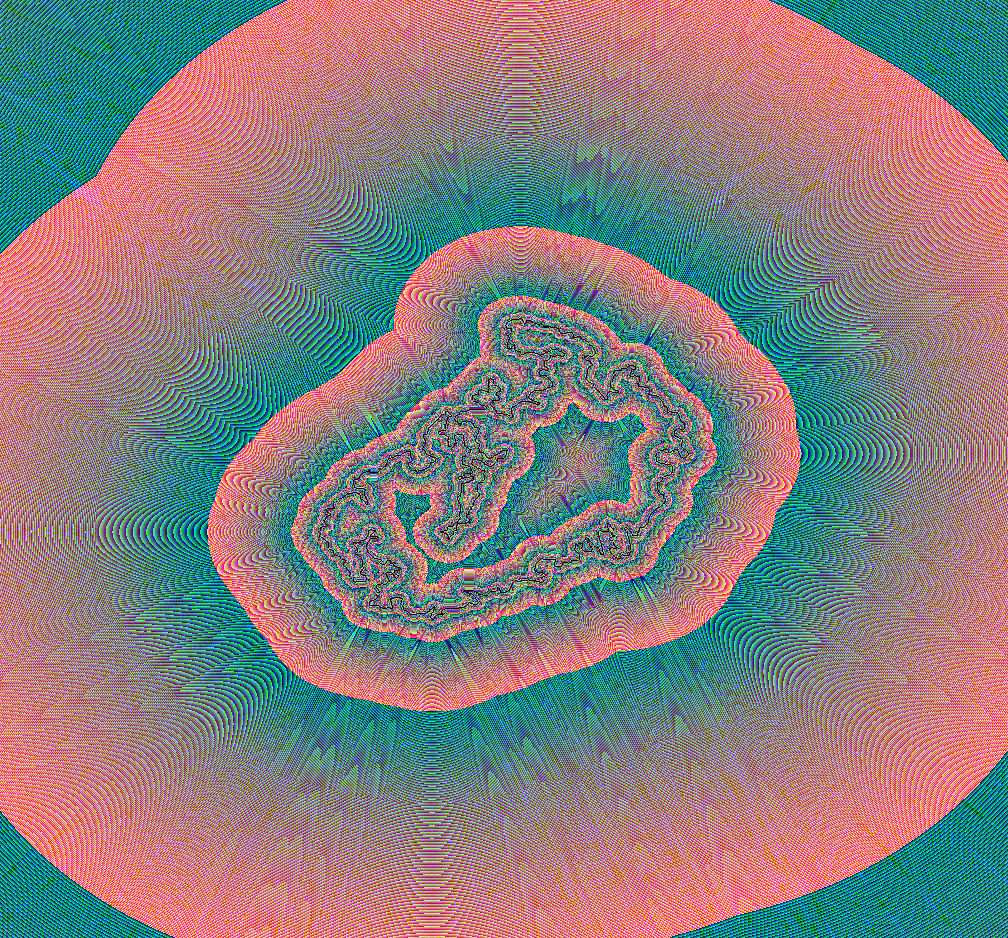

Supplement: S1 File — (ZIP) [file pone.0206565.s001.zip › Data used in methodology/the data used Fig 5/Dis.bmp]

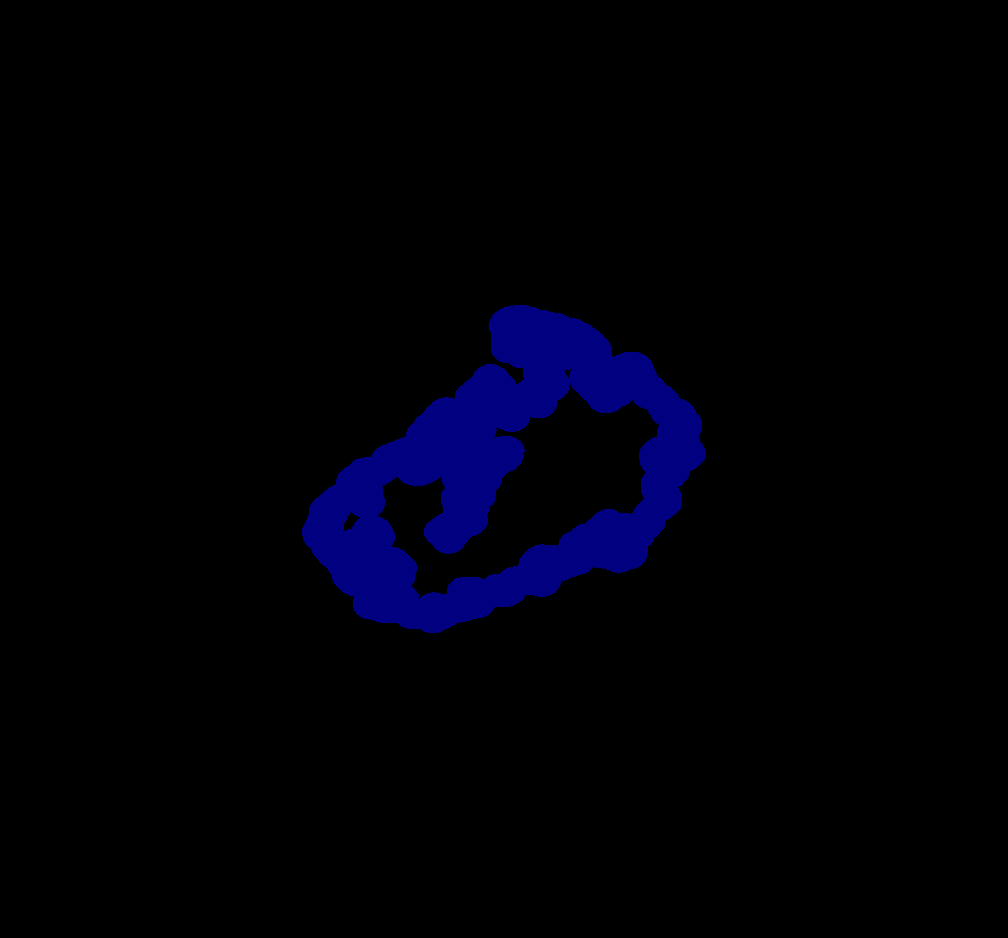

Supplement: S1 File — (ZIP) [file pone.0206565.s001.zip › Data used in methodology/the data used Fig 5/Ocp.bmp]
